# Twenty-Four-Month Safety and Effectiveness of TCD-17187 Drug-Coated Balloon for Treatment of Atherosclerotic Lesions in Superficial Femoral and Proximal Popliteal Artery

**DOI:** 10.1007/s00270-024-03747-4

**Published:** 2024-05-30

**Authors:** Yoshimitsu Soga, Osamu Iida, Shu-Ichi Seki, Daizo Kawasaki, Hitoshi Anzai, Hiroshi Ando, Tatsuya Nakama, Norihiko Shinozaki, Amane Kozuki, Masaharu Ishihara, Kazushi Urasawa, Satoru Toi, Hiroaki Tsujita, Kazuki Tobita, Kenji Ogata, Kazunori Horie, Naoki Hayakawa, Shinsuke Mori, Masahiko Fujihara, Takao Ohki, Kenichiro Yuba, Toshiaki Mano, Kenji Ando, Masato Nakamura, The TCD-17187 Japan Investigators, Yuji Ikari, Toshiro Shinke, Shigeru Saito, Yoshisato Shibata, Koichi Kishi

**Affiliations:** 1 Department of Cardiology, Kokura Memorial Hospital, Fukuoka, Japan; 2 Division of Cardiology, Cardiovascular Center, Osaka Police Hospital, Osaka, Japan; 3 Department of Medicine and Cardiology, Chikamori Hospital, Kochi, Japan; 4 Department of Cardiology, Morinomiya Hospital, Osaka, Japan; 5 Department of Cardiology, SUBARU Health Insurance Ota Memorial Hospital, Gunma, Japan; 6 Heart Center, Kasukabe Chuo General Hospital, Saitama, Japan; 7 Department of Cardiology, Tokyo Bay Medical Center, Chiba, Japan; 8 Department of Cardiology, Tokai University Hospital, Kanagawa, Japan; 9 Division of Cardiology, Osaka Saiseikai Nakatsu Hospital, Osaka, Japan; 10 Department of Cardiovascular and Renal Medicine, School of Medicine, Hyogo Medical University, Hyogo, Japan; 11 Cardiovascular Center, Tokeidai Memorial Hospital, Hokkaido, Japan; 12 Division of Minimally Invasive Treatment in Cardiovascular Medicine, Toho University Ohashi Medical Center, Tokyo, Japan; 13 Division of Cardiology, Department of Medicine, Showa University School of Medicine, Tokyo, Japan; 14 Cardiology and Catheterization Laboratories, Shonan Kamakura General Hospital, Kanagawa, Japan; 15 Department of Cardiology, Miyazaki Medical Association Hospital, Miyazaki, Japan; 16 Department of Cardiovascular Medicine, Sendai Kousei Hospital, Miyagi, Japan; 17 Department of Cardiovascular Medicine, Asahi General Hospital, Chiba, Japan; 18 Department of Cardiology, Saiseikai Yokohama City Eastern Hospital, Kanagawa, Japan; 19 Department of Cardiology, Kishiwada Tokushukai Hospital, Osaka, Japan; 20 Division of Vascular Surgery, Department of Surgery, Jikei University School of Medicine, Tokyo, Japan; 21 Department of Cardiology, Tokushima Red Cross Hospital, Tokushima, Japan; 22 Cardiovascular Center, Kansai Rosai Hospital, Hyogo, Japan

**Keywords:** Peripheral artery disease, Superficial femoral and proximal popliteal lesions, Endovascular therapy, Drug-coated balloon, Primary patency, Clinically driven target lesion revascularization

## Abstract

**Purpose:**

In the present trial, the 24-month safety and effectiveness of the TCD-17187 drug-coated balloon (DCB) for the treatment of atherosclerotic lesions in the superficial femoral artery (SFA) and proximal popliteal artery (PA) were evaluated in Japanese patients.

**Methods:**

This was a prospective, multicenter, core laboratory-adjudicated, single-arm trial. From 2019 to 2020, 121 patients with symptomatic peripheral artery disease were enrolled. The primary effectiveness outcome measure was primary patency. The safety outcome measure was the major adverse event (MAE) rate.

**Results:**

Age was 74.5 ± 7.3 years, and diabetes mellitus was present in 67.5%. Lesion length and reference vessel diameter (RVD) were 106.0 ± 52.6 mm and 5.2 ± 0.8 mm, respectively. Chronic total occlusion (CTO) and bilateral calcification rate (Grade 3 and 4 by peripheral arterial calcium scoring system (PACSS)) were 17.5% and 50.8%, respectively. The 24-month primary patency rate by duplex ultrasound was 71.3%, while freedom from clinically driven target lesion revascularization (CD-TLR) was 87.0%. The MAE rate was 13.2% and all events consisted of CD-TLR. There were no instances of device- or procedure-related deaths major amputations throughout the 24 months. Multivariate Cox proportional hazards regression analysis revealed significant differences associated with loss of primary patency in the following characteristics: CTO, restenotic lesion and RVD.

**Conclusion:**

This trial confirmed the safety and effectiveness of TCD-17187 DCB for atherosclerotic lesions of the SFA and/or proximal PA for up to 24 months.

**Level of Evidence:**

Level 3, Cohort study.

*Clinical Trial Registration*: URL: https://center6.umin.ac.jp/cgi-open-bin/ctr/ctr.cgi?function=brows&action=brows&recptno=R000038612&type=summary&language=J:Registration ID: UMIN000034122. Registration Date: September 13, 2018.

## Introduction

Therapy with DCB has emerged in the clinical setting and provides sustained clinical benefits in the treatment of FP lesions, compared to uncoated balloon angioplasty [1, 2]. This has had an impact on the field of endovascular treatment, leading to the widespread use of DCB as a common approach for FP lesions.

The performance of DCBs relies on their ability to transfer the drug efficiently into the vessel wall and facilitate sustained retention in the vessel tissue. These aspects are dependent on drug type/dose, excipients and coating technology, which generate differential effects on clinical outcomes. The specifications of the TCD-17187 DCB are as follows: paclitaxel (PTX) dose density of 3.2 µg/mm2, L-serine ethyl ester hydrochloride (L-SEE) as an excipient, and Unicoat™ technology (developed by Terumo Corporation) which facilitates uniform coating with PTX microcrystals. This is intended to ensure consistent drug delivery to the affected area and minimize peripheral embolism caused by peeling of the coating material.

However, even when DCB is used, long-term outcomes have been shown to be poor in patients with long lesions, severely calcified lesions, or diabetes [3, 4]. As already reported, in a Japanese patient population with many of these factors, the twelve-month primary patency rate of this study was 81.1%, while freedom from CD-TLR was 95.8%. The MAE rate at 12 months was 5.0%. There were no instances of device- or procedure-related deaths major amputations throughout the 12-month period. This study evaluated the 24-month safety and efficacy of TCD-17187 DCB.

## Materials and Methods

### Study Design

This was a prospective, multicenter, core laboratory-adjudicated, single-arm trial. The trial was independently monitored by a data safety monitoring board and clinical events committee (CEC) that reviewed and adjudicated all adverse events throughout the 24-month period following DCB treatment. Independent duplex ultrasound (DUS) (VasCore, Massachusetts General Hospital, Boston, MA, USA) and angiography (Beth Israel Deaconess Medical Center Cardiovascular Imaging *Core Laboratory*, Boston, MA, USA) core laboratories analyzed procedural and follow-up images. The trial was conducted in accordance with the Declaration of Helsinki, good clinical practice guidelines, and applicable laws.

### Study Population

Inclusion and exclusion criteria are summarized in the supplemental table. In brief, chronic symptomatic lower limb ischemia (classes 2–4 according to Rutherford’s classification) due to stenotic or non-stented restenotic lesions with a total lesion length ≤ 18 cm or totally occlusive lesions with a length of ≤ 10 cm involving the SFA and proximal PA was eligible. Prior to enrollment, written informed consent was obtained from all patients in accordance with the protocols approved by the institutional review boards at each investigational site.

### Description of TCD-17187 DCB

TCD-17187 (Terumo Corporation, Tokyo) has obtained CE but is not yet available in the market. It is coated with 3.2 μg/mm^2^ of PTX as an antiproliferative agent and a low-molecular-weight excipient L-SEE, which has hydrophobic groups that have affinity for PTX and hydrophilic groups that have affinity for water. The surface of the TCD-17187 DCB is uniformly coated with small PTX microcrystals, aiming an improvement in drug retention during balloon delivery and promotion of PTX release during balloon expansion by optimizing the balance between the two groups.

### Intervention Procedure and Follow-Up

Dual antiplatelet therapy (DAPT) was required prior to the index procedure. At the time of initial angiography, the status of in-flow and out-flow was evaluated to ascertain whether the patient was a suitable candidate for this trial. Heparin was administered at the beginning of the procedure, and an activated clotting time of 250 s was maintained throughout the procedure. Pre-dilatation was mandated, but no special PTA (percutaneous transluminal angioplasty) balloons including cutting or scoring balloons were allowed for pre-dilatation. DCB size was to have the same diameter as the reference vessel in a 1:1 balloon-to-vessel ratio. The recommend inflation time of the DCB was at least 180 s. Post-dilatation with a standard PTA balloon was allowed at the discretion of the operator. Provisional stenting was allowed only in the event of PTA failure after repeated and prolonged balloon inflations. PTA failure was defined as a residual stenosis ≥ 50% or major flow-limiting dissection (≥ Grade D on NHBLI). DAPT (100 mg/day aspirin with 75 mg/day clopidogrel or 3.75 mg/day prasugrel) was continued for at least 1 month after the index procedure. Follow-up was conducted at 30 days, 6 months, 1 year, and 2 years after the index procedure. The final follow-up was planned at 3 years postoperatively.

Demographic characteristics, comorbidities, ankle–brachial index (ABI), Rutherford classification, quality of life, angiographic lesion characteristics, and concomitant medications were recorded preoperatively. Intraoperatively, procedural details such as pre-dilatation and DCB use (size, dilatation pressure, dilatation time, angiographic procedural success, etc.) were evaluated. At each follow-up, we evaluated primary patency using DUS and assessed for any major adverse events (MAE) or other adverse events.

### Definitions

Primary patency was defined as core laboratory-assessed DUS peak systolic velocity ratio < 2.4 in the absence of CD-TLR. CD-TLR was defined as re-intervention at the target lesion due to recurrence of symptoms with ≥ 50% restenosis by core laboratory assessment, worsening of Rutherford classification, or decreasing ABI > 0.15, when compared with post-procedure baseline. Major amputation was defined as amputation of the limb above the ankle. Technical success was defined as residual stenosis in the treated segment of 30% or less without grade D or greater vessel dissection. Procedural success was defined as achieving technical success without the occurrence of a MAE during the index procedure. An MAE was defined as a composite of device- and procedure-related 30-day death, or an index limb major amputation and/or CD-TLR during follow-up.

### Study Outcome Measures

The primary efficacy outcome measure was primary patency after the index procedure up to 24 months. The secondary efficacy outcome measures comprised (1) freedom from CD-TLR, (2) change in ABI, (3) clinical improvement based on the Rutherford classification, and (4) the Walking Impairment Questionnaire (WIQ) score throughout the follow-up period. The primary safety outcome measure was the MAE rate. AEs were evaluated at 30 days, 6 months, 1 year, and 2 years. Death, repeat revascularization, and amputation were independently adjudicated by the CEC.

### Statistical Analysis

For effectiveness, the analysis dataset included all enrolled patients in the study except one patient who was adjudicated as having no ischemic vascular stenosis/occlusion. The Kaplan–Meier method was used to evaluate time-to-event data for primary patency at 24-month follow-up. In the exploratory data analysis, the hazard ratio was calculated using a univariate Cox regression model to examine patient, lesion, and procedure characteristics that may affect primary patency. For multivariate analysis with Cox regression model, the backward elimination was used with an elimination criterion of *p* value ≥ 0.1. Continuous variables were expressed as the mean ± standard deviation. Categorical variables were presented as counts and proportions.

Statistical analyses were performed using SAS software version 9.4 (SAS Institute, Cary, NC, USA).

## Results

### Baseline Characteristics and Intervention Procedure

From October 2019 to November 2020, 121 patients with symptomatic PAD due to SFA and/or proximal PA lesions were enrolled at 21 Japanese centers. Table 1 shows the baseline characteristics. As previously reported, age was 74.5 ± 7.3 years and males accounted for 73.3% (88/120) of the patient population. Notable comorbidities included diabetes mellitus in 67.5% (81/120) and kidney disease in 24.2% (29/120). History of any lower limb extremity revascularization before enrolling in this trial was present in 58.3% (70/120). In terms of severity of lower limb ischemia, the proportion of patients with claudication defined as Rutherford class 2 or 3 was 96.7% (116/120) of the population, and ABI before the index procedure was 0.72 ± 0.14. Lesion length and reference vessel diameter were 106.0 ± 52.6 mm and 5.2 ± 0.8 mm, respectively. Frequency of CTO, a lesion with bilateral calcification (Grade 3 and 4 on PACSS), and non-stented restenotic lesions were observed in 17.5% (21/120), 50.8% (61/120) and 10.8% (13/120), respectively. Table 2 shows the procedural characteristics. Post-dilatation was carried out in 9.2% (11/120) of patients after DCB dilatation, and the subsequent rate of provisional stenting was 0.8% (1/120, severe dissection) in this trial.

**Table 1:** Baseline patient and lesion characteristics

Characteristics^a^	All (*n* = 120)
*Clinical characteristics*	
Age (Years)	74.5 ± 7.3
Male, % (N)	73.3 (88)
Body mass index (kg/m^2^)	23.7 ± 3.7
Hypertension, % (N)	83.3 (100)
Dyslipidemia, % (N)	84.2 (101)
Diabetes mellitus, % (N)	67.5 (81)
Insulin-dependent diabetes mellitus, % (N)	19.2 (23)
Chronic kidney disease, % (N)	24.2 (29)
Heart failure, % (N)	5.8 (7)
Coronary artery disease, % (N)	51.7 (62)
Cerebrovascular disease, % (N)	15.8 (19)
*Smoking status, % (N)*	
Never	17.5 (21)
Past	53.3 (64)
Current	29.2 (35)
Treatment history of coronary artery disease, % (N)	40.0 (48)
Treatment history of cerebrovascular disease, % (N)	5.0 (6)
Treatment history of lower extremity arterial disease, % (N)	58.3 (70)
Endovascular therapy, % (N)	57.5 (69)
Surgical therapy, % (N)	0.8 (1)
ABI before index procedure	0.72 ± 0.14
*Rutherford class, % (N)*	
2	51.7 (62)
3	45.0 (54)
4	3.3 (4)
*Angiographic characteristics*	
De novo, % (N)^b^	89.2 (107)
Restenotic (non-stented), % (N)^b^	10.8 (13)
Popliteal involvement, % (N)^c^	1.7 (2)
*PACSS calcification, % (N)*^*c*^	
Grade 0	39.2 (47)
Grade 1	8.3 (10)
Grade 2	1.7 (2)
Grade 3	31.7 (38)
Grade 4	19.2 (23)
Lesion length (mm)^c, d^	106.0 ± 52.6
Chronic total occlusions, % (N)^c^	17.5 (21)
*TASC II classification, % (N)*^*c*^	
A	42.5 (51)
B	32.5 (39)
C	22.5 (27)
D	2.5 (3)
RVD (mm)^c^	5.2 ± 0.8
MLD (mm)^c^	1.1 ± 0.8
Diameter stenosis (%)^c^	78.3 ± 13.8

*ABI* ankle–brachial pressure index, *BTK* Below the knee, *MLD* Minimal lumen diameter, *N* numbers in category, *n* number of available values, *PACSS* Peripheral arterial calcium scoring system, *RVD* Reference vessel diameter, *TASC* Trans-Atlantic Inter-Society Consensus

^a^Continuous variables are presented as the means ± standard deviation; categorical variables are given as the percentage (counts)

^b^Site-reported

^c^Per lesion assessment evaluated by the core laboratory

^d^Normal-to-normal by core laboratory quantitative vascular analysis

**Table 2:** Procedural characteristics

Procedural characteristics^a^	All (*n* = 120)
Pre-dilatation, % (N)^b^	100 (120)
Balloon size of pre-dilatation (mm)^b^	5.5 ± 0.8
MLD after pre-dilatation (mm)^c^	3.5 ± 0.8
Diameter stenosis after pre-dilatation (%)^c^	31.8 ± 12.4
*Dissection after pre-dilatation, % (N)*^*c*^	
None	28.3 (34)
A-C	70.8 (85)
D-F	0.8 (1)
DCB size (mm)	5.7 ± 0.8
Transition time of DCB proceeding (sec)^b^	26.4 ± 16.9
Inflation time of DCB dilatation (sec)^b^	205.3 ± 48.1
Inflation pressure of DCB (atm)^b^	9.0 ± 2.0
The ratio of DCB size/reference vessel diameter^c^	1.11 ± 0.14
MLD after DCB dilatation (mm)^c^	3.8 ± 0.8
Diameter stenosis after DCB dilatation (%)^c^	27.8 ± 10.9
*Dissection after DCB dilatation, % (N)*^*c*^	
None	26.7 (32)
A-C	70.0 (84)
D-F	2.5 (3)
NA	0.8 (1)
Dissection length after DCB dilatation (mm)^c^	29.3 ± 20.5
Provisional stenting rate, % (N)^b^	0.8 (1)
Final diameter stenosis (> 30%), % (N)^c^	37.5 (45)
Achievement of TIMI 3 flow grade, % (N)^c,^ ^d^	97.5 (117)
Number of BTK run off^c^	1.4 ± 0.9
Technical success, % (N)	100.0 (120)
Procedural success, % (N)	100.0 (120)

*BTK* Below the knee, *DCB* Drug-coated balloon, *MLD* Minimal lumen diameter, *N* numbers in category, *n* number of available values, *NA* Not applicable, *TIMI* Thrombolysis in Myocardial Infarction Grade

^a^Continuous variables are presented as the means ± standard deviation; categorical variables are given as the count/sample (percentage)

^b^Site-reported

^c^Per lesion assessment evaluated by the core laboratory

^d^TIMI grade flow 3 indicates complete perfusion

### Efficacy Outcomes

**Fig. 1:**
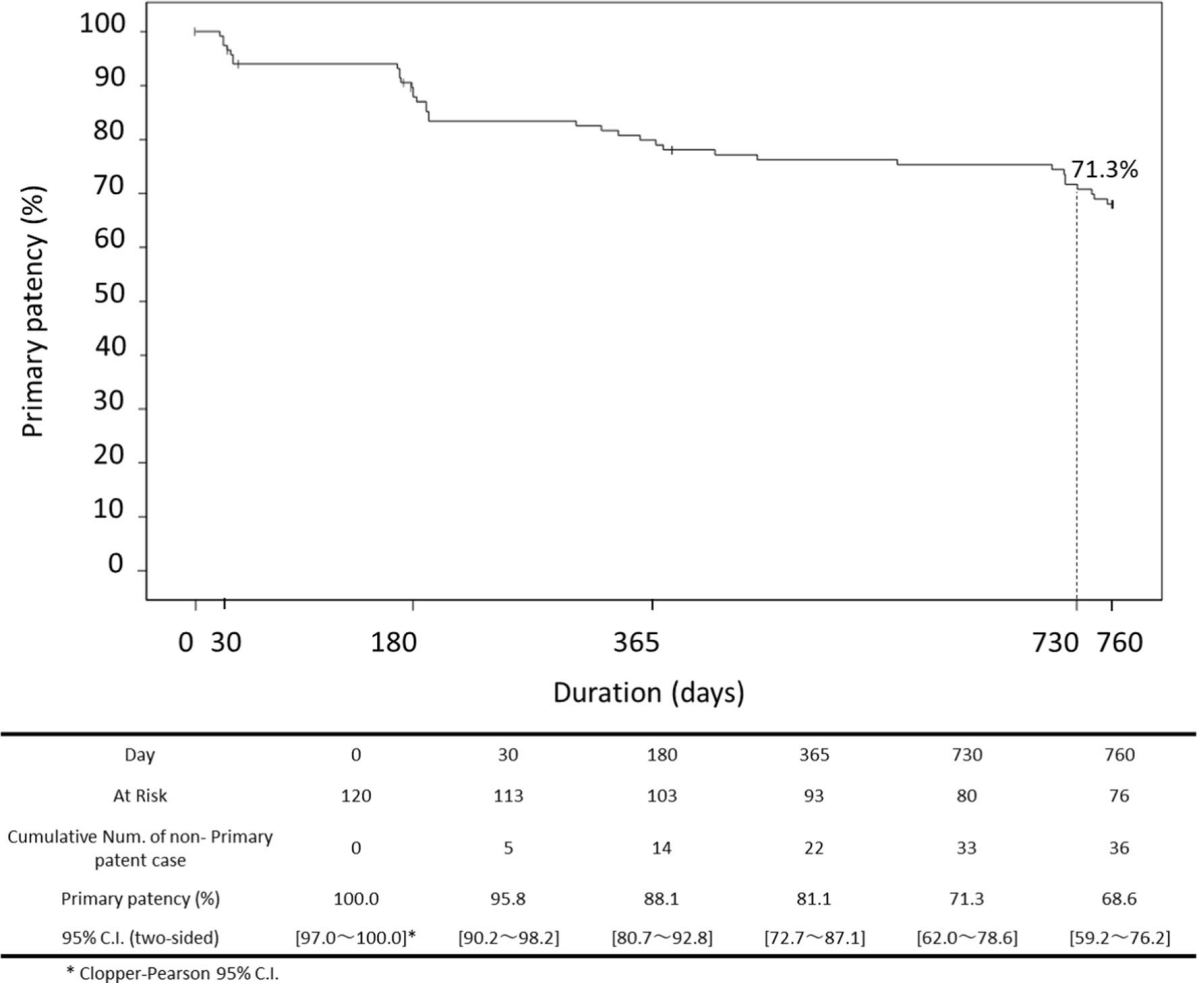
24-month outcomes for TCD-17187 drug-coated balloon. Kaplan–Meier estimates of primary patency

**Fig. 2:**
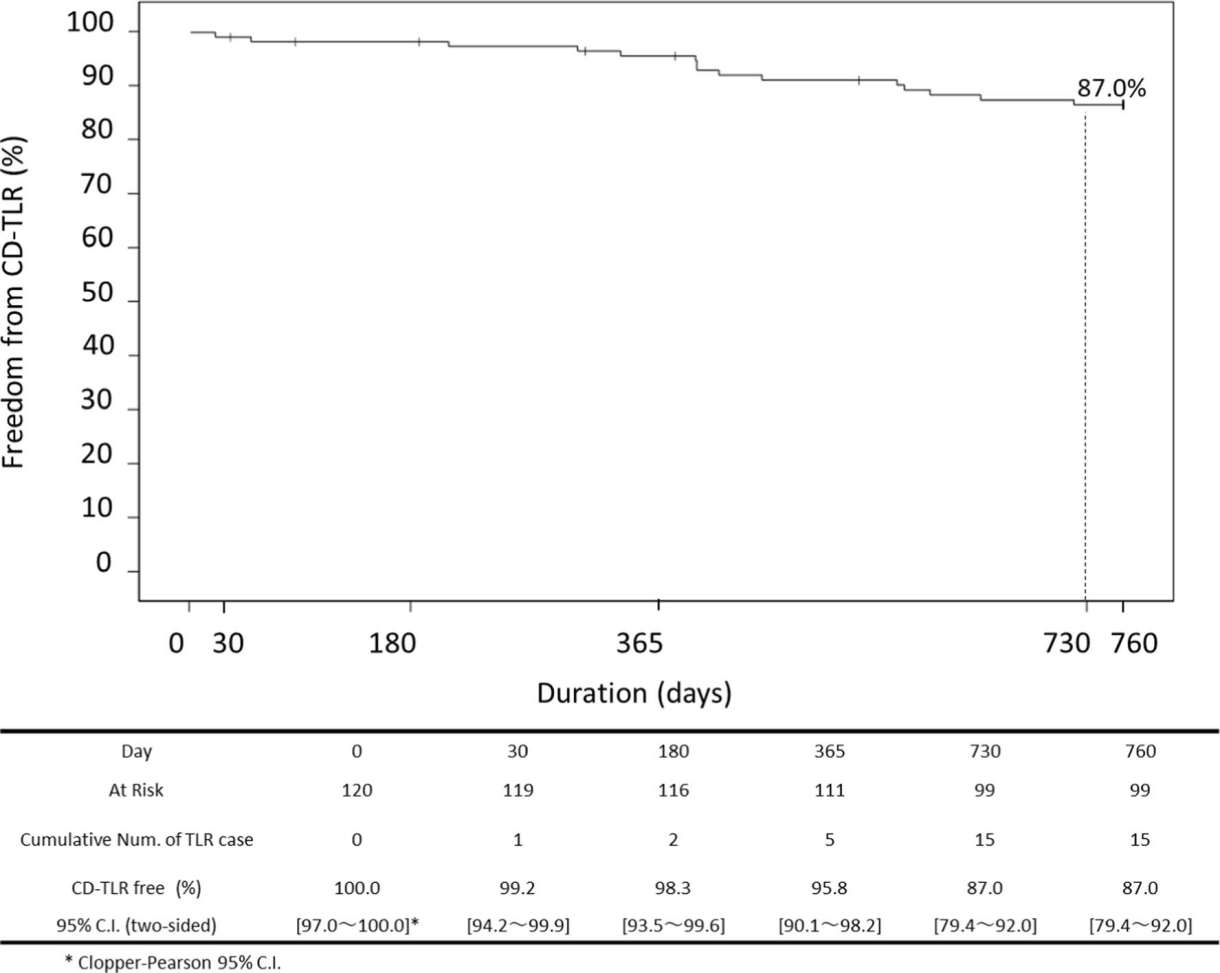
24-month outcomes for TCD-17187 drug-coated balloon. Kaplan–Meier estimates of freedom from restenosis and clinically driven lesion revascularization (CD-TLR)

Twenty-four-month primary patency (Fig. 1) and freedom from CD-TLR (Fig. 2) were 71.3% and 87.0%, respectively. A multivariate Cox proportional hazard regression analysis was used to identify predictive factors for loss of primary patency through 24 months. Kidney disease (adjusted hazard ratio [HR]: 0.32, 95% CI: 0.11–0.91, *p* = 0.033), CTO (adjusted HR: 2.59, 95% CI: 1.20–5.59, *p* = 0.015), restenotic lesion (adjusted HR: 2.69, 95% CI: 1.11–6.52, *p* = 0.028) and RVD (≥ 5 mm) (adjusted HR: 0.48, 95% CI: 0.25–0.95, *p* = 0.036) were significantly associated with rate of primary patency at 24 months (Table 3).

**Table 3:** Univariate Cox proportional hazards regression analysis over 2 years

Characteristics	Un-adjusted hazard ratio [95% confidence intervals] (*p* value)
*Clinical characteristics*	
Age (≥ 75 years)	0.79 [0.41, 1.51] (*p* = .470)
Female	0.75 [0.34, 1.64] (*p* = .469)
Dyslipidemia	2.00 [0.61, 6.51] (*p* = .251)
Diabetes mellitus	1.04 [0.52, 2.08] (*p* = .914)
Chronic kidney disease	0.34 [0.12, 0.96] (*p* = .042)
Treatment history of coronary artery disease	0.58 [0.29, 1.19] (*p* = .066)
Treatment history of lower extremity arterial disease	1.30 [0.66, 2.58] (*p* = .445)
Rutherford class (2, 3 versus 4)	2.54 [0.61, 10.58] (*p* = .201)
*Angiographic characteristics*	
Restenotic (non-stented)^a^	2.45 [1.07, 5.61] (*p* = .034)
PACSS calcification (0, 1, 2 versus 3, 4)^b^	1.04 [0.54, 1.99] (*p* = .915)
Lesion length (≥ 150 mm)^b^	1.13 [0.55, 2.35] (*p* = .741)
Chronic total occlusion^b^	2.09 [1.01, 4.33] (*p* = .048)
RVD (≥ 5 mm)^b^	0.47 [0.24, 0.91] (*p* = .025)
Presence of dissection at the end of index procedure^b^	0.78 [0.38, 1.58] (*p* = .487)
Final diameter stenosis (> 30%)^b^	0.67 [0.32, 1.39] (*p* = .278)

DCB, Drug-coated balloon; PACSS, Peripheral arterial calcium scoring system; RVD, Reference vessel diameter; TASC, Trans-Atlantic Inter-Society Consensus

^a^Site-reported

^b^Analyzed based on the results of the core laboratory

**Fig. 3:**
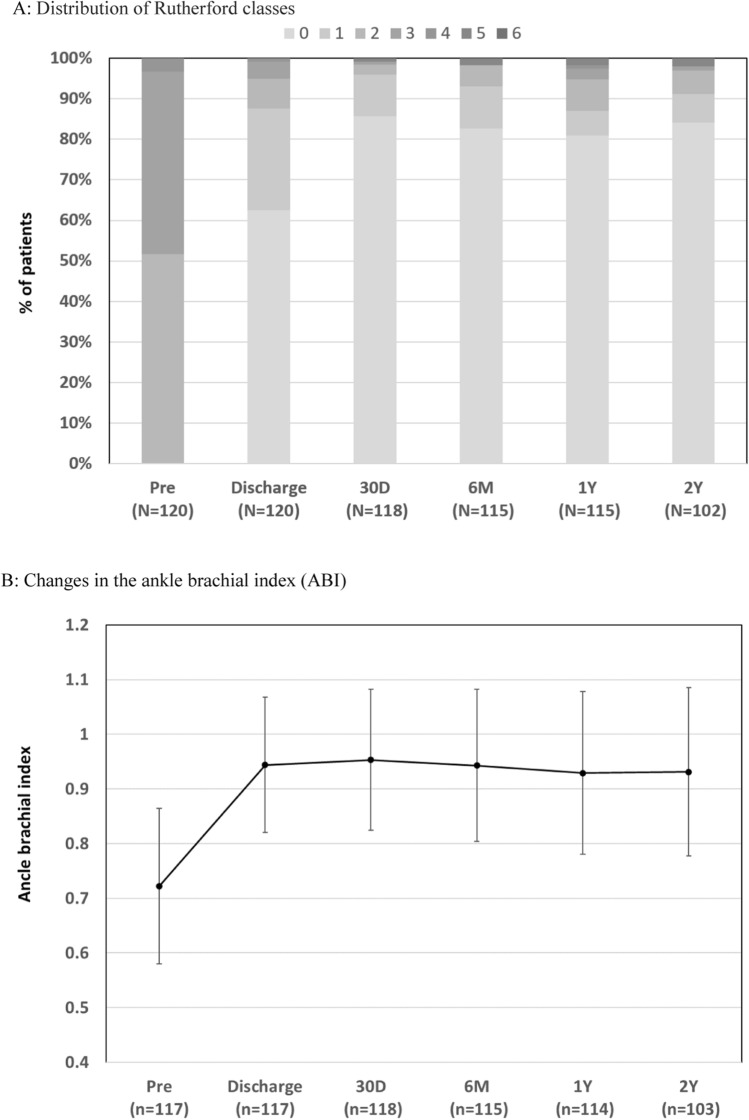

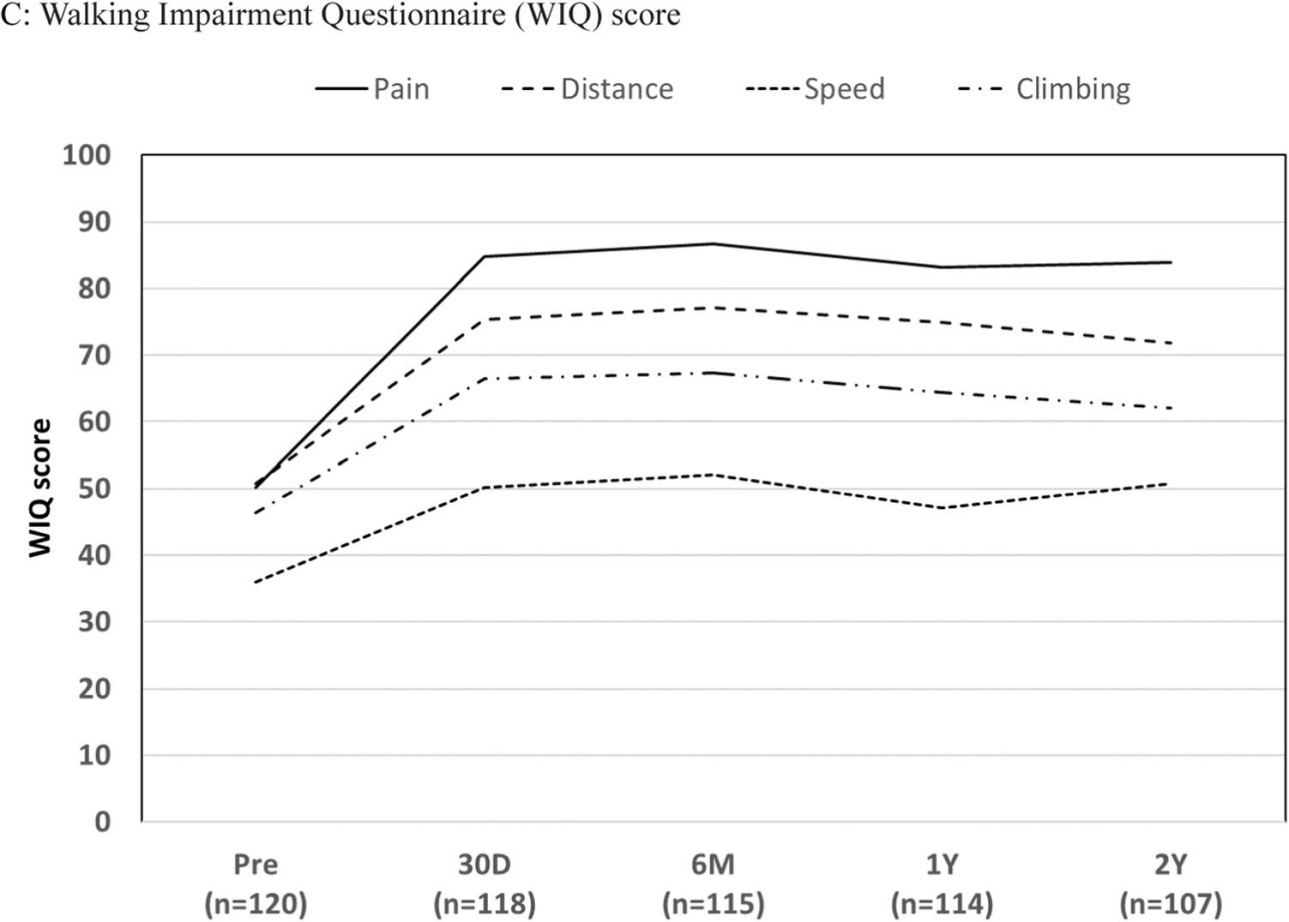
Clinical outcomes before and after treatment with TCD-17187 drug-coated balloon. **A** Distribution of Rutherford classes. **B** Changes in the ankle–brachial index (ABI). **C** Walking Impairment Questionnaire (WIQ) Score

Changes in the Rutherford classification, ABI, and WIQ score are shown in Fig. 3. Improvements in these quality of life indicators persisted for 24 months. Distribution of the Rutherford classes showed a trend toward improvement after the index procedure. At 24 months, 89.9% (98/109) of patients presented with symptoms classified as 0 or 1, with primary sustained clinical improvement.

### Safety Outcomes

The 24-month MAE was 13.2% (16/121) and consisted of CD-TLR alone; no target limb major amputation had been performed by 24 months and no 30-day deaths were observed (Table 4). During the 24-month follow-up, three patients died, but there were no deaths related to the procedure or investigational device.

**Table 4:** Safety outcomes

	0—30D (*n* = 121)	30D—6 M (*n* = 121)	6 M—1Y (*n* = 119)	1Y—2Y (*n* = 117)	Accumulation (*n* = 121)
MAE (Major Adverse Event), %	1.7 (2)	0.8 (1)	2.5 (3)	8.5 (10)	13.2 (16)
Death^a^, %	0 (0)	–	–	–	0 (0)
CD-TLR, %	1.7 (2)	0.8 (1)	2.5 (3)	8.5 (10)	13.2 (16)
Amputation^b^, %	0 (0)	0 (0)	0 (0)	0 (0)	0 (0)
All-cause death, %	0 (0)	0.8 (1)	1.7 82)	0 (0)	2.5^c^ (3)

Variables are given as the percentage (counts)

^a^Device or procedure related death within 30 days after index procedure

^b^Amputation made above the metatarsal line

^c^All deaths were unrelated to the study device and procedure

## Discussion

This trial aimed to assess the safety and effectiveness over a 24-month period. Compared to lesion characteristics in previous clinical trials of DCBs in Japan, in this trial, the prevalence of diabetes was higher, the average lesion length was longer, and the frequency of bilateral calcification (Grade 3and 4 on PACSS) was higher. Although these anatomical features have a major impact on loss of primary patency after EVT, [5–9] the 24-month primary patency in this study was comparable to that of the approved DCB and remained favorable [10, 11]. In terms of safety, there were 3 (2.5%) deaths, which was similar and low to the 24-month mortality rate (6.1%) of other premarket studies of DCBs conducted in Japan. [11]; all three deaths were non-cardiovascular death. No long-term increase in mortality was observed. Additionally, there were no deaths within 30 days or major amputation within 24 months, indicating that the device can be used safely up to 24 months.

To date, PTX has been the anti-restenosis drug of choice for the most commonly used DCBs owing to its highly lipophilic profile, fast tissue absorption, and long tissue retention, all of which have resulted in favorable long-term outcomes after treating FP lesions.

The COMPARE study, which evaluated the efficacy and safety of a high-dose (IN.PACT™) versus a low-dose (Ranger™) DCB in the treatment of FP lesions with DCB angioplasty, confirmed that 12-month clinical outcomes were comparable despite different coating dose and characteristics between the two DCBs [12]. This study also underscores the importance of both the excipient and the coating technology rather than the drug dose, as these two factors are crucial in efficient drug transfer into the vessel wall, sustained retention in the vessel tissue and achieving durable outcomes. The TCD-17187 DCB's novel technical design and characteristics achieved a good balance between drug retention during balloon delivery to the lesion and effective drug transit to the vessel wall. This optimization resulted in durable results without any increased risk of adverse events, including downstream effects adjudicated by core laboratory.

Multivariate analysis of variables for loss of primary patency revealed that presence of CTO, a restenotic lesion and a RVD (< 5 mm) were independent factors for loss of patency, which were consistent with the results obtained in other DCB studies [13–15]. Interestingly, factors related to patency were not evident at 1 year except for CTO, but long-term follow-up revealed that previously mentioned factors were involved. However, these factors were consistent with previous reports, with no specific factors. While other clinical and lesion factors already identified as positive predictors, such as lesion length, severity of vessel calcification, and TASC II classification, were not statistically significant.

These results potentially represent an insight into the specific features of the TCD-17187 DCB, but the sample size of this trial was limited and these findings need to be confirmed in real-world practice. Although there was a significant difference between restenotic and de novo lesions, 9 of the 13 restenotic lesions were restenotic lesions of other DCBs, and four were patent. Additionally and unexpectedly, multivariate analysis also revealed that presence of kidney disease was a protective factor for primary patency; as no specific reason was found in this study, this finding might be due to small sample size.

In terms of the occurrence of CD-TLR, the timing of CD-TLR occurrence deserves further attention and needs to be taken into consideration. As adjudicated by the blinded CEC, CD-TLR occurred in 15 patients, three of whom underwent reinterventions within six months of the initial procedure. Recent consensus based on the DISFORM study was that the occurrence of acute target lesion failure within 24 h or early restenosis at < 6 months was mainly attributable to post-angioplasty angiographic features including significant recoil and vessel dissection rather than any DCB effect [16]. Study candidates in this clinical trial were strictly screened by experienced physicians and all patients were angiographically evaluated; no severe recoil or vessel dissection was found. Taken together, defining procedural success as well as prediction of vessel patency based on the completion angiogram in the context of clinical trial remains a challenge.

### Study Limitations

This study was a single-arm study and had a limited sample size. The study population was restricted to Japanese patients, and thus, our findings may not generalize to other ethnic backgrounds. Whether or not the therapeutic effect of the TCD-17187 DCB is affected by racial differences needs to be clarified. Finally, in the current study, 70% of angiographic dissections in the final angiogram were mild-to-moderate, which has been considered a clinically acceptable feature without provisional stenting. However, three of the reinterventions were consequently performed within six months of the initial procedure. This finding may be attributable to the limitations of angiographic assessment for vessel dissection. Further investigation will be needed for a fundamental methodology for identifying vessel dissection based on functional assessment rather than visual assessment.

## Conclusions

This clinical trial demonstrated the 24-month safety and effectiveness of the TCD-17187 DCB in the treatment of atherosclerotic lesions in the SFA and/or proximal PA.
